# Doses of Nearby Nature Simultaneously Associated with Multiple Health Benefits

**DOI:** 10.3390/ijerph14020172

**Published:** 2017-02-09

**Authors:** Daniel T. C. Cox, Danielle F. Shanahan, Hannah L. Hudson, Richard A. Fuller, Karen Anderson, Steven Hancock, Kevin J. Gaston

**Affiliations:** 1 Environment and Sustainability Institute, University of Exeter, Penryn, Cornwall TR10 9EZ, UK; H.Hudson@exeter.ac.uk (H.L.H.); Karen.Anderson@exeter.ac.uk (K.A.); stevenhancock2@gmail.com (S.H.); K.J.Gaston@exeter.ac.uk (K.J.G.); 2 Zealandia, 31 Waiapu Road, Karori, Wellington 6012, New Zealand; danielleshanahan@gmail.com; 3 School of Biological Sciences, University of Queensland, St Lucia, Brisbane 4072, Australia; r.fuller@uq.edu.au

**Keywords:** depression, dose-response, exposure to nature, extinction of experience, nature dose, nature relatedness, physical behaviour, risk factors, social cohesion, self-assessment of health

## Abstract

Exposure to nature provides a wide range of health benefits. A significant proportion of these are delivered close to home, because this offers an immediate and easily accessible opportunity for people to experience nature. However, there is limited information to guide recommendations on its management and appropriate use. We apply a nature dose-response framework to quantify the simultaneous association between exposure to nearby nature and multiple health benefits. We surveyed ca. 1000 respondents in Southern England, UK, to determine relationships between (a) nature dose type, that is the frequency and duration (time spent in private green space) and intensity (quantity of neighbourhood vegetation cover) of nature exposure and (b) health outcomes, including mental, physical and social health, physical behaviour and nature orientation. We then modelled dose-response relationships between dose type and self-reported depression. We demonstrate positive relationships between nature dose and mental and social health, increased physical activity and nature orientation. Dose-response analysis showed that lower levels of depression were associated with minimum thresholds of weekly nature dose. Nearby nature is associated with quantifiable health benefits, with potential for lowering the human and financial costs of ill health. Dose-response analysis has the potential to guide minimum and optimum recommendations on the management and use of nearby nature for preventative healthcare.

## 1. Background

Exposure to nature brings a wide range of health benefits to humankind [1,2]. Population-level studies in developed countries have shown that people living in areas with higher levels of nature have improved mental [3], physical [4,5] and social [6] health, are more likely to undertake physical activity [7,8] and have a greater connection with nature [9,10]. Critically, these health benefits do not occur independently, but are delivered concomitantly as people spend time in nature. Research on determining the causal pathways by which these benefits are delivered is now increasingly well developed [11,12,13].

For most people, the nature around their home will provide their most common nature interactions [14], so it is likely critical for the provision of health benefits. This “nearby nature” offers an immediate and easily accessible opportunity for people to experience nature [15]. Such nature is provided by a combination of public and private green spaces. People will experience nearby nature as they consciously spend time in it, for example through gardening, and as they are subconsciously exposed to it as a by-product of other activities, such as walking to shops [1,16]. Private gardens are a major component of urban green space and contribute disproportionately towards nearby nature [17,18]. A significant number of private green spaces in the UK contain tall trees and vegetation [19] and are thus inevitably a central focus of people’s nearby nature experiences [20]. Gardens also provide locations where people can experience other multi-sensory components of nature that can be beneficial for health, such as sunlight and fresh air.

Given the wide availability of nearby nature, there is a huge opportunity to capitalise on it for health outcomes. Vegetation in the environment is associated with enhanced mental well-being [21,22,23], and short durations of exposure to natural environments deliver an immediate reduction in blood pressure [24] and greater feelings of mental restoration [25]. However, there is currently a dearth of information to guide recommendations on what kinds of nature and how frequently and how long people should spend in nature for improved health.

The nature dose-response framework [13,26,27,28] distinguishes three components of nature exposure, namely its intensity (quality and quantity), frequency and duration [13]. A dose-response approach can be used to develop minimum and optimal-dose recommendations to nature similar to those for physical activity [29]. Indeed, deconstructing nature dose is critical to identifying what environmental management interventions might be required to enhance the benefits that people receive from nature or precisely how people should alter their behaviour [13].

Here, we survey 1023 respondents in Southern England, UK, to quantify the link between five health outcomes and three measures of nearby nature dose. These five health domains all had plausible mechanistic pathways linking nature with health: mental health (self-reported depression) [21,22,23], physical health (self-assessment of general health) [24], social health (perceptions of social cohesion) [6], positive physical behaviour (level of physical activity) [30] and nature orientation (nature relatedness scale) [31]. Measures of nature dose were time spent in the garden in the previous week (frequency and duration of nature dose) and the quantity of vegetation surrounding the home (as a measure of dose intensity). Nature around the home commonly varies according to a suite of socio-demographic factors that also affect health (Table S1). Thus, we adjust for socio-economic and lifestyle covariates in our analyses to improve the detection of the nature benefits distinct from other potential confounding factors. We then use dose-response modelling to estimate the point at which the frequency and duration of visits to private green spaces and the quantity (intensity) of vegetation around the home altered the health outcomes measured here that could be represented in a binary fashion (depression).

## 2. Methods

### 2.1. Study Area and Survey Design

The present study formed part of the “Fragments, functions, flows and urban ecosystem services” project, looking at how the biodiversity in urban areas contributed to human health and well-being. It was conducted in the “Cranfield triangle” (52°07’N, 0°61’W), a region in southern England, UK, comprising the three adjacent towns of Milton Keynes, Luton and Bedford. This area has a human population of ca. 524,000 (2011 Census, UK) and occupies 157 km^2^. A lifestyle survey delivered online through a market research company (Shape the Future Ltd., Wadhurst, UK) was completed over a two-week period in May 2014 by 1023 adults enrolled in their survey database (see [32] for a full version of the survey). May is a period of reasonably mild weather when respondents were most likely to engage with nature around their home. During the survey period, there were maximum temperatures of 18.7 °C and minimum of 9.0 °C, with 39.6 mm of rainfall. The survey took approximately 20 min to complete, participants were self-selecting and were compensated with points that contributed towards a prize of their choosing. This research was conducted with approval from the Bioscience ethics committee of the University of Exeter (Project Number 2013/319). Participants provided written consent at the beginning of the online survey.

The survey collected socio-demographic and lifestyle variables that could influence health, including age, gender, the primary language spoken at home, personal annual income and highest formal qualification. As a potential confounder of recent nature exposure, we asked respondents relatively how much time they spent out of doors in the previous week (see Tables S2 and S3). Respondents were requested to provide a full UK postcode so that their neighbourhood could be characterised (at the scale of around 20 households).

### 2.2. Health Response Variables

Respondents provided self-reported information on five health domains:

- Mental health (binary): A measure of depression was generated based on the depression component of the short version of the Depression, Anxiety and Stress Scale (DASS 21; [33]). Scores were converted to a binary measure, where 0 indicates no depression and 1 indicates mild or worse depression (see Section 1 in Supplementary Materials). Proposed mechanisms for the delivery of these benefits include improved cognition in individuals with depression [34], reduced rumination and reduced neural activity in an area of the brain linked to the risk of mental illness [12].
- Physical health (ordinal): Respondents scored their own general health on a five-point scale from very poor to very good [35]. This scale is related to morbidity and mortality rates and is a strong predictor of health status and outcomes [36]. Proposed mechanisms behind benefit delivery include temperature regulation and pollution filtration by vegetation (reviewed by [27,37]).
- Social health (linear): Perceptions of social cohesion were estimated based on three previously developed scales that measure trust, reciprocal exchange within communities and general community cohesion ([38,39,40], see Section 2 in Supplementary Materials). The average score across questions for each scale was calculated, highest (4) to the lowest (0). Average scores were then summed to provide a scale from highest (12) to lowest (0). Appealing green spaces promote a sense of connection to the outside world that generalizes to most people; this allows enhanced social and community interactions, leading to improved perceptions of cohesion and well-being [41].
- Physical behaviour (Poisson): This is a self-reported indication of the number of days respondents exercised for a minimum of 30 min during the survey week (the duration recommended by the UK government) [42]. Appealing green spaces promote use [10] and willingness to travel greater distances for use [43]. Further, green exercise can enhance health benefits relative to built-up or indoor environments [30].
- Nature orientation (linear): Respondents provided a measure of their affective, cognition and experiential relationship with the natural world (nature relatedness scale) [31]. Responses were aggregated according to [31], with a higher score indicating a stronger orientation towards nature. Engagement with the natural world increases feelings of connection, unity or being part of the natural world, which has been linked to psychological health [44]. Indeed, increased nature connection has been associated with improved mental health [45] and subjective well-being [46,47].

### 2.3. Nature Dose

For each respondent, we generated three measures of the dose of nearby nature: frequency and duration (time spent in private green space) and intensity (quantity of neighbourhood vegetation cover). The frequency of nature dose was estimated based on the respondents’ self-reported frequency of more than ten minutes spent in their own garden in the last week. Respondents selected from: never, <once, once, 2–3 days, 4–5 days, 6–7 days. The duration of nature dose was estimated based on self-reported total time spent in the garden within the last week. Respondents selected from: no time, 1–30 min, >30 min–1 h, >1–3 h, >3–5 h; >5–7 h, >7–9 h, 9 or more hours. The mid-points of the selected categories were used for statistical analysis*.* People experience nature from time spent in the garden through both intentional interactions, such as gardening, and incidental interactions as they immerse themselves in multiple multi-sensory nature experiences while engaged in non-nature-based activities [1]. The intensity of nature dose was measured as neighbourhood vegetation cover of ≥0.7 m in height within a 250-m buffer around the centroid of each respondent’s postcode. This is the distance that was considered to influence what can be seen or experienced from a person’s home on a day-to-day basis. Only those respondents who provided a full UK postcode were included in the analyses involving this variable (*n* = 474). The vegetation cover maps used here were derived from an airborne hyperspectral data and LiDAR; full details of their development are provided in Section 3 in Supplementary Materials. In brief, vegetation was separated from non-vegetation by those pixels (2 m resolution) with a Normalised Difference Vegetation Index >0.2 [48]. Pixels with an NDVI > 0.2 and a mean height of first return more than 0.7 m above the ground were marked as tall vegetation. Heights from discrete return LiDAR are well-known to produce biased results over vegetation [49], and so, this 0.7 m threshold may have represented a more variable vegetation threshold height. All data extraction and analysis was performed in QGIS (v2.6; [50] and in R (v3.2; [51]).

### 2.4. Statistical Analysis

We examined the relationships between each health response variable and potential predictors, including socio-demographic variables, self-assessment of health, physical activity, social cohesion and nature relatedness (where the predictor variable was not also a response variable). We used generalized linear models (binomial) for depression, cumulative link models for self-assessment of health, linear regression for social cohesion and nature relatedness and Poisson regression models for physical activity. The frequency and duration of nature doses are inextricably linked (duration could only be measured where respondents visited a green space at least once a week). Consequently, these variables were correlated (Spearman’s rank test correlation of 0.67); to avoid multicollinearity we generated four predictor model sets for each health response: (i) socio-demographic variables; (ii) socio-demographic variables plus frequency of nature exposure; (iii) socio-demographic variables plus duration of nature exposure; and (iv) socio-demographic variables plus intensity of nature exposure. We used the MuMIn’ package [52] to produce all subsets of models based on the global model and rank them based on **Δ**AICc. To be 95% sure that the most parsimonious models were contained within the best supported model set, we retained all models where **Δ**AICc < 6 [53]. We then calculated averaged parameter estimates and standard errors using model averaging [54].

One of the response variables was binary (depression), which allowed us to model the dose-response relationship with nature exposure [55]. Ordinal (physical health) and continuous (social health, physical behaviour and nature relatedness) response variables do not lend themselves easily to this approach, because there is no threshold where a score is “good” or “bad”. We estimated the relative odds that an individual will have depression given their specific risk factors (e.g., age) and varying levels of nature exposure. We first ran a series of logistic regression models to test the association between depression and the predictor variables plus varying levels of each of the three categories of nature dose in turn. We used only those predictor variables that were significant in the first analysis, and using existing evidence where possible, we transformed each into a binary risk factor conveying “high” (1) versus “low” (0) risk (Table S4). We also transformed each of the nature dose variables into binary risk factors by setting incrementally higher thresholds of exposure. For example, when testing the relationship between frequency of exposure and depression, we tested a series of variables where each person’s frequency of visits was categorized as less than (1) or ≥once per week (0) and less than (1) or ≥2–3 times per week (0; Table S4). For each dose, we then identified the point at which the health gains were first recorded as better than the null model on a plot of dose versus the odds ratio for use in the analysis described below (i.e., the confidence interval did not overlap with an odds ratio of one).

The population average attributable fraction was calculated to estimate the proportion of depression cases in the population attributable to each of the predictor variables (or risk factors) [56]. Each risk factor was removed sequentially from the population by classifying every individual as low risk. The probability of each person having depression was then calculated, where the sum of all probabilities across the population was the adjusted number of disease cases expected if the risk factor was not present. The attributable fraction was calculated by subtracting this adjusted number of cases from the observed number of cases. The risk factors were removed in every possible order, and an average attributable fraction from all analyses was obtained.

## 3. Results

**Figure 1:**
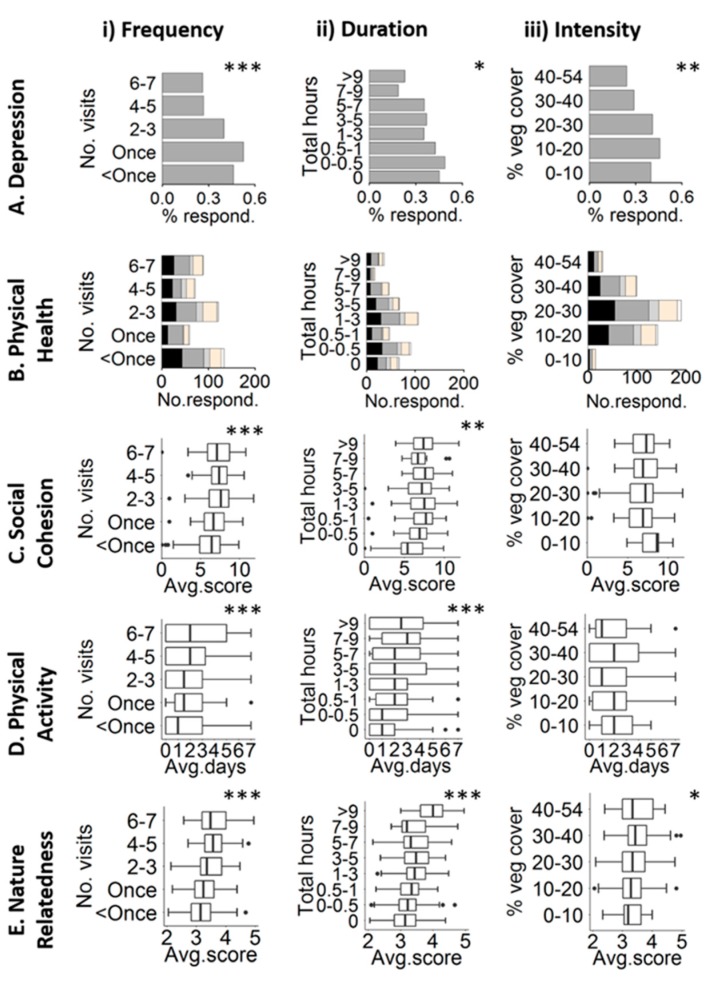
The relationship between health responses (**A**−**E**) and nature exposure, comprising (**i**) frequency of garden visits, (**ii**) duration of garden visits and (**iii**) neighbourhood nature intensity, measured as the percentage vegetation cover within a 250 m buffer of the centre of the respondents’ postcodes. We show significant relationships within the regression models outlined in Table 1, and error bars are standard errors. Physical health (B) shows the number of respondents for each nature dose that had very good (white), good (light grey), average (medium grey), poor (dark grey) and very poor (black) self-reported health. Significance (* *p* <0.05; ** *p* < 0.01; *** *p* < 0.001).

The survey respondents tended to be younger, but otherwise were of a similar demographic to those in the local population (Table S2). Across the respondents’ neighbourhoods, there was an average vegetation cover of 24% (±9.1% SD) and built cover of 55.7% (±14.2% SD), with most respondents having access to private gardens (91.4%). We found that four of the health outcomes, namely depression, perceptions of social cohesions, levels of physical activity and nature orientation, improved with an increasing frequency and duration of exposure to nearby nature (i.e., there was a positive association with perceptions of social cohesion, levels of physical activity and nature orientation and a negative association with levels of depression; Table 1; Figure 1). We also found that a greater intensity of nature exposure was associated with lower levels of mild or worse depression and higher levels of nature relatedness (Table 1; Figure 1). These relationships held even after accounting for potential covariates. We did not find any relationship between nearby nature and self-reported physical health (Table 1; Figure 1). Respondents who spent relatively less time out of doors in the survey week were more likely to have depression and to have worse physical behaviour, while respondents who spent relatively more time outdoors had increased nature relatedness.

**Figure 2:**
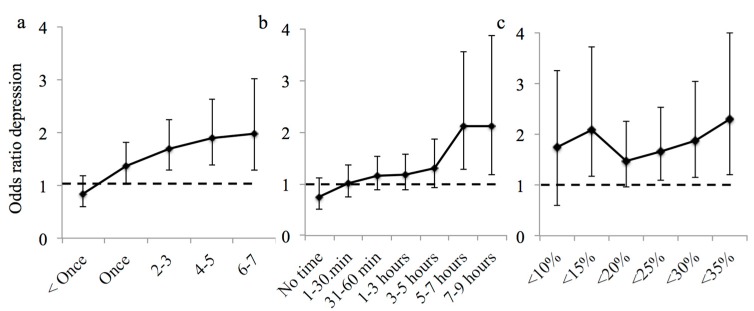
Dose-response graphs showing the adjusted odds ratio from logistic regression of depression for (**a**) incrementally increasing frequency of visits of ten minutes or more to a private green space; (**b**) total duration of time spent in private green space in the past week and (**c**) percentage neighbourhood vegetation cover. The 95% confidence intervals are shown. An odds ratio above one indicates that an individual is more likely to have depression where the nature dose is not met.

The odds of having mild or worse depression were lower than the null model when the frequency of garden visits was once a week or greater, with further incremental gains until an optimum of 4−5 times a week after which subsequent benefits to mental health were limited (Table 2; Figure 2a). There was a minimum and optimum threshold at five or more hours in the duration of the total time spent in the garden, after which the levels of depression rapidly decreased (Table 2; Figure 2b). The dose-response relationship was less consistent for nature intensity. The levels of depression were lower in people who lived in neighbourhoods with 15% vegetation cover followed by no effect at 20% cover, then further incremental gains in lower rates of depression at 25%, until 35% vegetation cover was met (Table 2; Figure 2c). The optimal dose-intensity did not appear to have been met in this study (Figure 2c).

## 4. Discussion

We demonstrate that nature close to the home is associated with quantifiable benefits to population health. We found measurably better mental health, social health, positive physical behaviour and nature orientation with greater frequency and duration of time spent in nearby nature. We also showed lower levels of depression and greater nature orientation in people who live in greener neighbourhoods. However, we found no relationship with self-reported physical health. 

We carried out a dose-response analysis to identify the point at which exposure to nature was associated with a lower incidence of depression in the surveyed population. The key challenge for the cross-sectional design used in this study is the potential existence of a circular feedback loop, where people with depression might avoid going outdoors. Thus, a lower dose of nature might be an outcome, rather than a cause of the observed depression. However, this type of dose-response analysis should not be considered in isolation; rather, it adds a thread of evidence to the growing body of literature demonstrating a link between mental health outcomes and nature dose (as per Hill’s criteria for causality; [57]). As such, if the link is in fact casual, our dose-response analysis suggests that up to 5% and 27% of depression cases within our survey population could be prevented if all city residents spent 10 min or more a week in their garden or five hours or more in total, respectively; or, if neighbourhood vegetation is managed to a minimal level of 15% cover, it could prevent up to a further 5% of depression cases. If scaled-up to the urban population, this suggests that behavioural interventions that encourage exposure to nearby nature and even minimum recommended levels of neighbourhood greening could have considerable impact on population health. The potential savings associated with improving nature exposure would be significant given that in 2007, it was estimated that depression cost the English economy £7.5 billion in health costs and lost workdays [58].

We found that across four self-reported health outcomes, the frequency of nature exposure was a stronger predictor than the duration of exposure. This has implications for the design of health interventions. It has been recognised in the sport sciences that short frequent exposures are a time-efficient strategy to induce health outcomes [59]. Thus, people may be able to gain their necessary nature dose while going about their daily activities, such as walking to shops, or spending time in a room with a view of nature.

The dose-response analysis showed that all three types of exposure to nearby nature had positive associations with survey population levels of depression. The dose-response relationship observed for frequency (≥1 garden visit a week) and intensity (≥25% vegetation cover) is considered to provide some evidence of causality according to Hill’s criterion (i.e., reduced levels of depression with increasing increments of dose) [57]. Visiting gardens 4−5 times a week appeared to create an optimal response and was associated with 17% lower levels of depression in the survey population; further increases in dose had limited further benefits. An optimal dose had yet to be reached for intensity, because few respondents lived in neighbourhoods with >35% tree cover, and so, the standard error was too great to detect a reliable signal. A higher duration of exposure was also associated with lower levels of depression, with a minimum and optimum threshold of significantly lower levels of depression beyond five hours of exposure. There is evidence that experiencing nature improves mood in people with depression [34], and multiple and multi-sensory elements doubtless contribute to these improvements through a variety of mechanistic pathways. Respondents who spent relatively less time out of doors in the survey week were more likely to report worse depression. Intriguingly, this suggests that relative nature experience may be a contributing factor. The type of nature exposure and the severity of depression may have important implications for the mechanistic pathway through which nature affects mental health, and thus, nature dose recommendations could be tailored for the specific needs of people with poor mental health.

Population-level studies have shown that increased green space has been associated with lower mortality from cardio-vascular disease [4] and enhanced general and self-reported health [60,61]. However, other studies found no association between green space cover and mortality, or even increases in mortality at the citywide scale [62,63]. This study further suggests that physical health benefits may be location specific depending on risk factors prevalent in individual cities.

We quantified the relationship between spending time in nearby nature and social health, showing that visiting the garden just once a week or spending up to even 30 min a week in the garden is associated with significantly greater perceptions of social cohesion between neighbours. Green space provides opportunities for more frequent encounters between neighbours that create and strengthen social ties, leading to increased social cohesion [64,65]. Subjective experiences of the views of nature from home, the quality of nature and the amount of time spent in nature have all been linked to perceiving one’s community as linked and cohesive [41], illustrating that nearby nature provides a variety of benefits to community health through multiple pathways. 

The frequency and duration of time spent in nearby nature were important predictors of physical activity. Although we did not assess the type of physical activity, the strong relationship does suggest that either spending time in nearby nature is a strong motivator for people to engage in physical activity or that more active people spend more time in nearby nature (reviewed by [66]). Either way, these green spaces not only provide important locations to exercise, but there is robust evidence that they also enhance the benefits of physical activity to both physical [66] and mental health [25], which may further motivate people to exercise more.

For the first time, we have quantified the relationships between doses of nature close to the home and nature orientation. Our analysis shows that once a minimal dose threshold is met, there are consistently higher levels of nature orientation with further incremental increases in dose. Our results support previous research that showed a positive relationship between time spent in the garden with nature orientation [9]. Interestingly, people who spent relatively more time out of doors had higher nature relatedness, suggesting that the recent doses of nature may contribute towards shaping nature orientation. Maintaining nature around the home may therefore be critical for both health and biological conservation, because nature orientation has been associated with improved life happiness [46,47], reduced anxiety [45] and environmental behaviour [67].

This study used a cross-sectional design, which inevitably has both advantages and limitations. The main advantage is that this allows the simultaneous analysis of multiple risk factors. The limitation is that this design cannot definitively establish a cause-effect relationship; however, these pathways are becoming increasingly well developed by other studies [11,12,13]. This study also relied on self-reported data, which may lead to common method bias. Thus, additional studies using more objective health indicators, including hair cortisol or heart rates, might be needed. Health is a complex issue with multiple drivers, and although we controlled for key socio-economic covariates known to influence health, the impact of life events, such as family emergencies, is difficult to control for. The low R^2^ (see Table 1) indicates a low predictive power; however, within the variables tested, exposure to green space was a significant predictor of improved health. This study was conducted over a two-week period in May when the benefits of nature are predicted to be greatest and the levels of depression may be lower [68]. Nonetheless, experiences of nature vary greatly across the year, and understanding how this variation influences nature doses and the associated health benefits is an important direction for future research. Further, studies unpicking the influence of nature exposure on health relative to factors associated with time out of doors, such as exposure to sunlight and vitamin D absorption, are required. Finally, the benefits of contact with nature vary across socio-economic groups, cultures and environments, and as such, caution must be applied when drawing conclusions applicable to broader populations. Future research needs to establish how the health benefits from nature vary across these different axes.

## 5. Conclusions

Nearby nature offers huge potential as an easily accessible and cost-effective approach to illness prevention. Close partnership among ecologists, health scientists and health practitioners, along with town planners and landscape architects, will be essential to capitalise on this opportunity. This will produce cost effective health policies that flexibly meet the needs of a range of communities. We demonstrate that dose-response threshold analysis has great potential in providing a framework guiding recommendations for green space management and use.

## Figures and Tables

**Table 1:** The relationship between five health responses and (i) socio-demographic only; (ii) plus frequency; (iii) plus duration and (iv) plus intensity.

Variables	Mental Health	Physical Health	Social Health	Physical Behaviour	Nature Relatedness
Model (i)	**R^2^ = 0.12**	**^#^**	**R^2^ = 0.15**	**R^2^ = 0.06**	**R^2^ = 0.14**
Intercept	**4.62 ** **(** 0.90) ***	NA	3.40 (0.62) ***	−0.76 (0.25) **	2.71 (0.09) ***
Age	−0.23 (0.03) ***	−0.11 (0.03 ) ***	−0.05 (0.03) *	−0.03 (0.01) **	0.05 (0.01) ***
Gender_female	−0.16 (0.15)	−0.26 (0.13) *	−0.01 (0.13)	−0.05 (0.04)	0.10 (0.03) **
Children in home	−0.02 (0.07)	−0.05 (0.06)	0.06 (0.06)	0.06 (0.02) **	0.01 (0.01)
Language at home	0.27 (0.20)	0.08 (0.17)	0.26 (0.17)	−0.07 (0.06)	0.05 (0.04)
Work days per week	−0.08 (0.04)	0.08 (0.03) *	−0.02 (0.04)	0.02 (0.01)	−0.02 (0.01) *
Income	−0.03 (0.04)	0.13 (0.03) ***	0.18 (0.03) ***	0.02 (0.02)	−0.02 (0.01) **
Frequency of 30-min exercise	−0.02 (0.04)	0.19 (0.03) ***	0.11 (0.03) ***	NA	0.04 (0.01) ***
Social cohesion	−0.01 (0.04)	0.20 (0.03) ***	NA	0.05 (0.01) ***	0.04 (0.01) ***
Nature relatedness	−0.28 (0.26)	−0.12 (0.14)	0.73 (0.14) ***	0.26 (0.05) ***	NA
Education (highest qual.)					
A-level	0.2 (0.20)	0.41 (0.16) *	0.18 (0.17)	−0.11 (0.06)	0.02 (0.04)
Undergraduate	−0.10 (0.25)	0.47 (0.18) **	0.17 (0.18)	−0.04 (0.06)	0.04 (0.04)
Postgraduate	0.01 (0.25)	1.05 (0.21) ***	0.38 (0.21)	−0.09 (0.07)	0.08 (0.05)
Self-assessment health					
Poor	−1.01 (0.59)	NA	−0.05 (0.44)	−0.04 (0.18)	−0.06 (0.01)
Average	−1.66 (0.56) **	NA	0.18 (0.40)	−0.04 (0.16)	−0.10 ( 0.10)
Good	−2.55 (0.59) ***	NA	0.81 (0.40) *	0.29 (0.16)	−0.10 (0.10)
Very good	−2.58 (0.57) ***	NA	1.29 (0.41) **	0.44 (0.16) **	−0.10 (0.10)
Relative time outdoors					
About the same	−0.83 (0.19) ***	−0.07 (0.16)	−0.16 (0.16)	0.15 (0.06) ***	0.02 (0.04)
More time	−1.15 (0.22) ***	−0.05 (0.18)	−0.22 (0.18)	0.28 (0.07) ***	0.11 (0.04) **
Model (ii)	**R^2^ = 0.13**	**^#^**	**R^2^ = 0.17**	**R^2^ = 0.06**	**R^2^ = 0.17**
Nature exposure frequency exposure	−0.2 (0.05) ***	0.03 (0.05)	0.23 (0.05) ***	0.09 (0.02) ***	0.07 (0.01) ***
Model (iii)	**R^2^ = 0.13**	**^#^**	**R^2^ = 0.16**	**R^2^ = 0.06**	**R^2^ = 0.18**
Nature exposure duration	−0.06 (0.03) *	0.01 (0.02)	0.07 (0.02) **	0.03 (0.01) ***	0.04 (0.01) ***
Model (iv)	**R^2^ = 0.17**	**^#^**	**R^2^ = 0.15**	**R^2^ = 0.08**	**R^2^ = 0.14**
Nature exposure intensity	−0.04 (0.01) **	0.01 (0.01)	0.01 (0.01)	0.004 (0.003)	0.004 (0.002) *

**^#^** No pseudo R^2^ available for ordinal regression. Model averaged coefficients are shown with the standard error in brackets, and the pseudo R^2^ is McFadden’s. Positive coefficients indicate that rates of depression are higher and that physical activity, social cohesion, physical activity and nature relatedness increased. Boldface indicates statistical significance (* *p* <0.05; ** *p* < 0.01; *** *p* < 0.001).

**Table 2:** Odds ratio and average attributable fraction of having depression where specific risk factors are present.

Variable	Risk Factor	Odds Ratio (95% CI)	Average Population Fraction
Age	Higher risk < 46 years	2.94	0.41
		(1.96, 4.41)	
Self-assessment of physical health	Higher risk < average health	3.64	0.07
		(2.25, 5.90)	
Relative time outdoors	Higher risk < less time outdoors	2.51	0.08
		(1.76, 3.56)	
Frequency of exposure	Higher risk < once per week	1.36	0.05
		(1.02, 1.81)	
Duration of exposure	Higher risk < five hours per week	2.12	0.27
		(1.27, 3.54)	
Intensity of exposure	High risk < 15% vegetation cover	2.09	0.05
		(1.17, 3.72)	

An odds ratio above 1 indicates that depression is more likely to be present where the risk factor is present.

## Supplementary Materials

The following are available online at www.mdpi.com/1660-4601/14/2/172/s1, Table S1: Socio-demographic variables and the categorisation used in the analysis, Table S2: The socio-economic distribution of all respondents (*n* = 1023) and of the subset who provided postcodes, thus allowing their geographical location to be modelled (shown in brackets; *n* = 473). We also show the demographics of the local population (Census 2011), Table S3: Spearman rank correlations between socio-demographic variables, Table S4: Binary risk factors for each covariate. For those predictor variables that were statistically significant in Table 1, we transformed each into a binary risk factor conveying “high” (1) versus “low” (0) risk. We used existing evidence where possible. We also transformed each of the nature dose variables into binary risk factors by setting incrementally higher thresholds of exposure.

## References

[1] Keniger L.E., Gaston K.J., Irvine K.N., Fuller R.A. (2013). What are the benefits of interacting with nature?. Int. J. Environ. Res. Public Health.

[2] Hartig T., Mitchell R., de Vries S., Frumkin H. (2014). Nature and health. Annu. Rev. Public Health.

[3] White M.P., Alcock I., Wheeler B.W., Depledge M.H. (2013). Would you be happier living in a greener urban area? A fixed-effects analysis of panel data. Psychol. Sci..

[4] Mitchell R., Popham F. (2008). Effect of exposure to natural environment on health inequalities: An observational population study. Lancet.

[5] Donovan G.H., Butry D.T., Michael Y.L., Prestemon J.P., Liebhold A.M., Gatziolis D., Mao M.Y. (2013). The relationship between trees and human health evidence from the spread of the emerald ash borer. Am. J. Prev. Med..

[6] Kingsley J.Y., Townsend M. (2006). “Dig In” to social capital: Community gardens as mechanisms for growing urban social connectedness. Urban Policy Res..

[7] Sugiyama T., Francis J., Middleton N.J., Owen N., Giles-Corti B. (2010). Associations between recreational walking and attractiveness, size, and proximity of neighborhood open spaces. Am. J. Public Health.

[8] Lee C., Ory M.G., Yoon J., Forjuoh S.N. (2013). Neighborhood walking among overweight and obese adults: Age variations in barriers and motivators. J. Community Health.

[9] Lin B.B., Gaston K.J., Fuller R.A., Wu D., Bush R., Shanahan D.F. (2017). How green is your garden? Urban form and socio-demographic factors influence yard vegetation, visitation, and ecosystem service benefits. Landsc. Urban Plan..

[10] Shanahan D.F., Cox D.T.C., Fuller R.A., Hancock S., Lin B.B., Anderson K., Bush R., Gaston K.J. (2017). Variation in experiences of nature across a gradient of tree cover in compact and sprawling cities. Landsc. Urban Plan..

[11] Hanski I., von Hertzen L., Fyhrquist N., Koskinen K., Torppa K., Laatikainen T., Karisola P., Auvinen P., Paulin L., Nakela M.J. (2012). Environmental biodiversity, human microbiota, and allergy are interrelated. Proc. Natl. Acad. Sci. USA.

[12] Bratman G.N., Hamilton J.P., Hahn K.S., Daily G.C., Gross J.J. (2015). Nature experience reduces rumination and subgenual prefrontal cortex activation. Proc. Natl. Acad. Sci. USA.

[13] Shanahan D.F., Fuller R.A., Bush R., Lin B.B., Gaston K.J. (2015). The health benefits of urban nature: How much do we need?. BioScience.

[14] Miller J.R., Hobbs R.J. (2002). Conservation where people live and work. Conserv. Biol..

[15] Lachowycz K., Jones A.P. (2013). Towards a better understanding of the relationship between greenspace and health: Development of a theoretical framework. Landsc. Urban Plan..

[16] Pretty J. (2004). How nature contributes to mental and physical health. Spiritual. Health Int..

[17] Gaston K.J., Fuller R.A., Loram A., MacDonald C., Power S., Dempsey N. (2007). Urban domestic gardens (XI): Variation in urban wildlife gardening in the United Kingdom. Biodivers. Conserv..

[18] Goddard M.A., Dougill A.J., Benton T.G. (2010). Scaling up from gardens: Biodiversity conservation in urban environments. Trends Ecol. Evol..

[19] Gaston K.J., Warren P.H., Thompson K., Smith R.M. (2005). Urban domestic gardens (IV): The extent of the resource and its associated features. Biodivers. Conserv..

[20] Freeman C., Dickinson K.J.M., Porter S., van Heezik Y. (2012). “My garden is an expression of me”: Exploring householders’ relationships with their gardens. J. Environ. Psychol..

[21] Ulrich R.S., Altman I., Wohlwill J.F. (1983). Aesthetic and affective response to natural environment. Behavior and the Natural Environment.

[22] Kaplan S. (1995). The restorative benefits of nature—Toward an integrated framework. J. Environ. Psychol..

[23] Berman M.G., Jonides J., Kaplan S. (2008). The cognitive benefits of interacting with nature. Psychol. Sci..

[24] Hartig T., Evans G.W., Jamner L.D., Davis D.S., Garling T. (2003). Tracking restoration in natural and urban field settings. J. Environ. Psychol..

[25] Barton J., Pretty J. (2010). What is the best dose of nature and green exercise for improving mental health? A multi-study analysis. Environ. Sci. Technol..

[26] Jiang B., Li D., Larsen L., Sullivan W.C. (2016). A dose-response curve describing the relationship between urban tree over density and self-reported stress recovery. Environ. Behav..

[27] Shanahan D.F., Lin B.B., Bush R., Gaston K.J., Dean J.H., Barber E., Fuller R.A. (2015). Toward improved public health outcomes from urban nature. Am. J. Public Health.

[28] Sullivan W.C., Frumkin H., Jackson R.J., Chang C.Y. (2014). Gaia meets Asclepius: Creating healthy places. Landsc. Urban Plan..

[29] Powell K.E., Paluch A.E., Blair S.N. (2011). Physical activity for health: What kind? How much? How Intense? On top of what?. Annu. Rev. Public Health.

[30] Richardson E.A., Pearce J., Mitchell R., Kingham S. (2013). Role of physical activity in the relationship between urban green space and health. Public Health.

[31] Nisbet E.K., Zelenski J.M., Murphy S.A. (2009). The nature relatedness scale linking individuals’ connection with nature to environmental concern and behavior. Environ. Behav..

[32] Shanahan D.F., Bush R., Gaston K.J., Lin B.B., Dean J., Barber E., Fuller R.A. (2016). Health benefits from nature depend on dose. Sci. Rep..

[33] Lovibond S.H., Lovibond P.F. (1995). Manual for the Depression Anxiety Stress Scales.

[34] Berman M.G., Kross E., Krpan K.M., Askren M.K., Burson A., Deldin P.J., Kaplan S., Sherdell L., Gotlib I.H., Jonides J. (2012). Interacting with nature improves cognition and affect for individuals with depression. J. Affect. Disord..

[35] Subramanian S.V., Huijts T., Avendano M. (2010). Self-reported health assessments in the 2002 World Health Survey: How do they correlate with education?. Bull. World Health Organ..

[36] Idler E.L., Benyamini Y. (1997). Self-rated health and mortality: A review of twenty-seven community studies. J. Health Soc. Behav..

[37] Salmond J.A., Tadaki M., Vardoulakis S., Arbuthnott K., Coutts A., Demuzere M., Dirks K.N., Heaviside C., Lim S., Macintyre H. (2016). Health and climate related ecosystem services provided by street trees in the urban environment. Environ. Health.

[38] Sampson R.J., Raudenbush S.W., Earls F. (1997). Neighborhoods and violent crime: A multilevel study of collective efficacy. Science.

[39] Bullen P., Onyx J. (1998). Measuring Social Capital in Five Communities in NSW—A Practitioner's Guide.

[40] Sampson R.J., Morenoff J.D., Earls F. Reciprocated Exchange. http://dcyfernetsearch.cehd.umn.edu/sites/default/files/PsychometricsFiles/Sampson−Reciprocated%20Exchange%20(Ages%2018−older).pdf.

[41] Weinstein N., Balmford A., Dehaan C.R., Gladwell V., Bradbury R.B., Amano T. (2015). Seeing community for the trees: The links among contact with natural environments, community cohesion, and crime. Bioscience.

[42] (2011). Start Active, Stay Active: A Report on Physical Activity from the Four Homecountries’ Chief Medical Officers. https://www.gov.uk/government/uploads/system/uploads/attachment_data/file/216370/dh_128210.pdf..

[43] Giles-Corti B., Johnson M., Knuiman M., Donovan R. (2005). Increasing walking—How important is distance to, attractiveness, and size of public open space?. Am. J. Prev. Med..

[44] Feral C.-H. (1998). The connectedness model and optimal development: Is ecopsychology the answer to emotional well-being?. Hum. Psychol..

[45] Martyn P., Brymer E. (2014). The relationship between nature relatedness and anxiety. J. Health Psychol..

[46] Capaldi C.A., Dopko R.L., Zelenski J.M. (2014). The relationship between nature connectedness and happiness: A meta-analysis. Front. Psychol..

[47] Zelenski J.M., Nisbet E.K. (2014). Happiness and feeling connected: The distinct role of nature relatedness. Environ. Behav..

[48] Liang S., Kong J.A. (2004). Quantitative Remote Sensing of Land Surfaces.

[49] Hancock S., Disney M., Muller J.-P., Lewis P., Foster M. (2011). A threshold insensitive method for locating the forest canopy top with waveform lidar. Remote Sens. Environ..

[50] Quantum GIS Development Team Quantum GIS Geographic Information System v2.6. Open Source Geospatial Foundation Project. http://qgis.osgeo.org.

[51] R Development Core Team (2016). R: A Language and Environment for Statistical Computing.

[52] Bartoń K. (2015). MuMIn: Multi-Model Inference. R Package Version 1.13.4. http://CRAN.R-project.org/package=MuMIn.

[53] Richards S.A. (2005). Testing ecological theory using the information-theoretic approach: Examples and cautionary results. Ecology.

[54] Burnham K.P., Anderson D.R. (2002). Model Selection and Multimodel Inference: A Practical Information-Theoretic Approach.

[55] World Health Organization Global Strategy on Diet, Physical Activity and Health: Physical Inactivity: A Global Public Health Problem. http://www.who.int/dietphysicalactivity/factsheet_inactivity/en/.

[56] Rueckinger S., von Kries R., Toschke A.M. (2009). An illustration of and programs estimating attributable fractions in large scale surveys considering multiple risk factors. BMC Med. Res. Methodol..

[57] Hill A.B. (1965). Environment and disease-association or causation. Proc. R. Soc. Med..

[58] Das J., Do Q.-T., Friedman J., McKenzie D., Scott K. (2007). Mental health and poverty in developing countries: Revisiting the relationship. Soc. Sci. Med..

[59] Gibala M.J., Little J.P., van Essen M., Wilkin G.P., Burgomaster K.A., Safdar A., Raha S., Tarnopolsky M.A. (2006). Short-term sprint interval versus traditional endurance training: Similar initial adaptations in human skeletal muscle and exercise performance. J. Physiol..

[60] Maas J., Verheij R.A., Groenewegen P.P., de Vries S., Spreeuwenberg P. (2006). Green space, urbanity, and health: How strong is the relation?. J. Epidemiol. Community Health.

[61] Groenewegen P.P., van den Berg A.E., Maas J., Verheij R.A., de Vries S. (2012). Is a green residential environment better for health? If So, why?. Ann. Assoc. Am. Geogr..

[62] Richardson E., Pearce J., Mitchell R., Day P., Kingham S. (2010). The association between green space and cause-specific mortality in urban New Zealand: An ecological analysis of green space utility. BMC Public Health.

[63] Richardson E.A., Mitchell R., Hartig T., de Vries S., Astell-Burt T., Frumkin H. (2012). Green cities and health: A question of scale?. J. Epidemiol. Community Health.

[64] Kuo F.E., Sullivan W.C., Coley R.L., Brunson L. (1998). Fertile Ground for community: Inner-city neighborhood common spaces. Am. J. Community Psychol..

[65] Sullivan W.C., Kuo F.E., DePooter S.F. (2004). The fruit of urban nature—Vital neighborhood spaces. Environ. Behav..

[66] Shanahan D.F., Franco L., Lin B.B., Gaston K.J., Fuller R.A. (2016). The benefits of natural environments for physical activity. Sports Med..

[67] Restall B., Conrad E. (2015). A literature review of connectedness to nature and its potential for environmental management. J. Environ. Manag..

[68] Harmatz M.G., Well A.D., Kawamura K.Y., Rosal M., Ockene I.S. (2000). Seasonal variation of depression and other moods: A longitudinal approach. J. Biol. Rhythm..

