# Supplementary material for: Doses of Nearby Nature Simultaneously Associated with Multiple Health Benefits
Source: Int J Environ Res Public Health. 2017 Feb 9;14(2):172. doi: 10.3390/ijerph14020172 (PMC5334726; doi:10.3390/ijerph14020172)
Supplement: Supplementary file 1 [file ijerph-14-00172-s001.pdf]

Supplemental Material: Doses of Nearby Nature Simultaneously Associated with Multiple Health Benefits

Daniel T. C. Cox, Danielle F. Shanahan, Hannah L. Hudson, Richard A. Fuller, Karen Anderson, Steven Hancock and Kevin J. Gaston

1. Development of Depression Measure

We used the depression component of the Depression, Anxiety and Stress Scale (DASS 21) included in the urban lifestyle questionnaire (taken from [1] and reproduced here for ease of reference). Respondents were asked to rate how much each of seven statements applied to them over the past week. Answers were given on a four-point scale from: did not apply to me at all; applied to me to some degree, or some of the time; applied to me to a considerable degree, or a good part of the time; applied to me very much, or most of the time; "I couldn't seem to experience any positive feelings at all", "I found it difficult to work up the initiative to do things", "I felt that I had nothing to look forward to", I felt down-hearted and blue", "I was unable to become enthusiastic about anything", "I felt I wasn't worth much as a person", "I felt life was meaningless". The severity of depression was then rated by summing the scores (normal, 0–4; mild, 5–6; moderate, 7–10; severe, 11–13; extremely severe, 14+). Finally, in the nature dose, analysis predictors are treated as "risk factors", an established practice in population epidemiology [2,3]. We therefore converted the score to a binary measure, as those without depression and those with mild or worse depression.

2. Development of Social Cohesion Measure

We generated estimates of each respondent's perception of social cohesion using three sets of questions that provided an indication of trust, reciprocal exchange within communities and general community cohesion. The first set was a social cohesion and trust scale developed by [4]. Respondents were asked how strongly they agreed (selecting from "don't know", "disagree strongly", "disagree", "agree", "agree strongly") that "People in this community are willing to help their neighbours", "This is a close-knit community", "People in this community can be trusted", "People in this community generally don't get along with each other" and "People in this community do not share the same values". Items were scored from 0–4; low scores indicated poor social cohesion with "don't know" scoring zero (as it indicated no knowledge of the community in which a person lived), through to "agree strongly", which was coded the highest at four. The last two statements were reverse coded. The second set was adapted from the reciprocated exchange scale developed by [5]. Respondents answered "don't know", "never", "rarely", "sometimes", "often" (scored 0–4 respectively) to six items, specifically "About how often do you and people in your community do favours for each other?", "When a neighbour is not at home how often do you and other neighbours watch over their property?", "About how often do you and people in your community ask each other advice about things such as child rearing or job openings?", "About how often do you and people in your community visit in each other's homes or on the street?", "About how often do you and people in your community have parties or other get-togethers?", "About how often do you and people in your community spend leisure time together going out for dinner, to the movies, to a sporting event etc.?" The third set provided a general measure of social capital using components from [6], with respondents answering "don't know", "not at all", "not often", "sometimes" or "yes, definitely" (scaled 0–4, respectively) to six questions. These were "Do you feel safe walking alone down your street after dark?", "Do you feel valued by society?", "Do you feel there are opportunities to have a real say on issues that are important to you?", "Can you get help from friends, family and neighbours when needed?"

"Do you help out a local group as a volunteer?", "Do you think multiculturalism makes life in your area better?" For all three sets of questions, an average score was generated, and higher scores

indicated greater natural capital. Finally, to provide an overall estimate of social cohesion, the scores from the three scales were averaged for inclusion in the analysis.

3. Characterisation of Neighbourhood Urban Form

The urban form of the neighbourhood of each respondent who provided a postcode was characterised using airborne hyperspectral data (Eagle spectrometer) and LiDAR (Leica ALS50-II) data collected by the Natural Environment Research Council (NERC) Airborne Research and Survey Facility (ARSF) aircraft in July and September 2012. The Normalized Difference Vegetation Index (NDVI) was calculated from the hyperspectral data using a red band focused at 570 nm and a near infrared band focused at 860 nm with a spatial resolution of 2 m. Histograms of NDVI were examined and a threshold of 0.2 identified as being suitable to separate vegetated (NDVI ≥ 0.2) from non-vegetated (NDVI < 0.2) pixels [7]. The LiDAR data were used in discrete return mode, with up to four returns per laser pulse. The laser point density was between one point per 25 cm^2 and one point per 2 m^2, depending on the flight line overlap. The lastools software [8] "lasground" function was used to find ground returns within the LiDAR point cloud. Pixels (2-m resolution) with an NDVI greater than 0.2 and a mean height of first return more than 0.7 m above the ground were marked as tall vegetation. Heights from discrete return LiDAR are well known to produce biased results over vegetation [9], and so, this 0.7-m threshold may have represented a more variable vegetation threshold height; since that bias is most usually an underestimation, it could correspond to taller vegetation (up to 1.7 m tall). All data extraction and analysis were performed in QGIS (v2.6; [10]) in R (v3.2; [11]).

Table S1. Socio-demographic variables and the categorisation used in the analysis.

Variable	Categorisation	Description
Age	Age (ordinal)	Respondents selected from 11 age bands: 18–20 years, then increasing in increments of 5 years until respondents were >70 years
Gender	Gender (categorical)	Female or male
Children in home	Number of children living at home (count)	Respondents were asked the number of children under 16 years who were living at home
Language	Primary language spoken at home (categorical)	Respondents speak a language other than English at home (no or yes)
Education	Highest formal education (categorical)	Highest qualification (selected from 4 categories equivalent to: general certificate of secondary education; A-levels; bachelor's degree; postgraduate degree)
Relative time outdoors	Recent nature experience (factor)	Respondents selected from: less time, about the same time, more time
Income	Personal annual income (ordinal)	Respondents selected from eight brackets: No income; £1–£199 a week (£1–£10,399 per year); £200–£299 a week (£10,400–£15,599 per year); £300–£399 (£15,600–£20,799 per year); £400–£599 a week (£20,800–£31,199 per year); £600–£799 a week (£31,200–£41,599 per year); £800–£9,999 a week (£41,600–£51,999 per year); >£1000 a week (>£52,000)
Work days per week	Work days per week (count)	Respondents provided a self-reported indication of the number of days a week they work. The resulting count variable was between 0 and 7

Table S2. The socio-economic distribution of all respondents (*n* = 1023) and of the subset who provided postcodes, thus allowing their geographical location to be modelled (shown in brackets; *n* = 473). We also show the demographics of the local population (Census 2011).

Variable	Percentage of Respondents	Demographic of Local Population *
Age (in years)	18–20 years: 6% (7%);<br>21–25 years: 12% (9%);<br>26–30 years: 15% (12%);<br>31–35 years: 18% (16%);<br>36–40 years: 11% (9%);<br>41–45 years: 10% (10%);<br>46–50 years: 9% (10%);<br>51–55 years: 6% (7%);<br>56–60 years: 6% (10%);<br>61–65 years: 3% (6%);<br>66–70 years: 3% (2%);<br>>70 years: 1% (2%)	11–19 years: 11.4%<br>20–39 years: 23.2%<br>40–59 years: 28.9%<br>60–74 years: 15%<br>74+ years: 7.8%
Gender	Male: 47% (49%);<br>Female: 53% (51%)	Male: 49%<br>Female: 51%
Children in home	No children: 42% (50%);<br>One child: 36% (29%);<br>Two children: 15% (15%);<br>Three children: 5% (3%);<br>Four children: 1.5% (2%);<br>Five children: 0.6% (0.6%)	No children: 56%<br>Non-dependent children: 9.8%<br>Dependent children:34.2%
Language (English primary language spoken at home)	No 15% (15%);<br>Yes 85% (85%)	No: 15%<br>Yes: 85%
Education	GCSE: 20% (2.3%);<br>A-level: 37% (37.1%);<br>Bachelors: 27% (29.2%);<br>Post-graduate: 16% (10.5%)	No qualification:16.8%<br>GCSE: 28.1%<br>A-level: 11.9%<br>Undergraduate & above: 34.8%<br>Other (including foreign qualifications: 4.9%
Relative time outdoors	Less time: 19%<br>About the same: 54%<br>More time: 27%	Unknown in local population
Income	No income: 3.8% (3.4%);<br>£1–£10,399 per year: 12.0% (11.6%);<br>£10,400–£15,599 per year: 10.7% (9.5%);<br>£15,600–£20,799 per year: 15.5% (10.7%);<br>£20,800–£32,199 per year: 25.4% (17.6%);<br>£32,200–£41,599 per year: 15.7% (14%);<br>£41,600–£51,999 per year: 9.2% (7.1%);<br>>£52,000 per year: 7.5% (18.5%)	No income: 1.2%<br>£1–15,588: 31%<br>£15,600–£31,199: 37%<br>>31,200: 30.8%
Work days per week	0 day: 11.1% (18.3%);<br>1 days: 1.8% (1.6%);<br>2 days: 3.3% (3.2%);<br>3 days: 5.6% (5.7%);<br>4 days: 9.8% (9.3%);<br>5 days: 56.2% (51.9%);<br>6 days: 9.4% (7.1%);<br>7 days: 2.5% (2.6%)	15 h or less a week: 9.3%<br>16–30 h a week: 18.3%<br>31–48 h a week: 60.3%<br>49 h or more a week: 12.2%

* Note: the format of data collection in the 2011 Census differed from this survey, and so, it was not possible to compare demographics directly.

Table S3. Spearman rank correlations between socio-demographic variables.

Variable	1	2	3	4	5	6	7
1. Age							
2. Children in home	−0.22						
3. Ethnicity	−0.09	0.08					
4. Work days per week	−0.21	0.06	0.01				
5. Income	0.03	0.1	0.01	0.25			
6. Frequency of 30 min exercise	−0.09	0.1	−0.01	0.08	0.11		
7. Social cohesion	−0.07	0.07	−0.03	0.06	0.21	0.21	
8. Nature relatedness	0.24	−0.02	0.02	−0.15	−0.06	0.14	0.15

Table S4. Binary risk factors for each covariate. For those predictor variables that were statistically significant in Table 1, we transformed each into a binary risk factor conveying "high" (1) versus "low" (0) risk. We used existing evidence where possible. We also transformed each of the nature dose variables into binary risk factors by setting incrementally higher thresholds of exposure.

Variable	Conversion to Binary Risk Factor
Age	The prevalence of mood disorders begins to decline around 45 years [12]. We therefore created a binary risk factor, at which above 45 years, the risk of having poor mental health was (0) and below was (1).
Self-assessment of health	There is a higher prevalence of poor mental health in people with poor physical health (e.g., [13]). We created a binary risk factor at which the risk of having poor mental health was (0) in people with average to very good health and (1) in people with poor to very poor health.
Relative time outdoors	Respondents were considered at higher risk of poor mental health if they spent less time out of doors than usual in the previous week (1). If respondents spent the same, or more time out of doors than usual, they were considered at low risk of poor mental health (0).
Frequency of exposure	Respondents were considered to be at a higher risk of poor mental health if the frequency of visits were not met: less than (1) or ≥ once per week (0); less than (1) or ≥2–4 times per week (0); less than (1) or ≥4–5 times per week (0); less than (1) or ≥6–7 times per week (0).
Duration of exposure	Respondents were considered to be at a higher risk of poor mental health if duration of visits were not met: less than (1) or ≥1–30 min per week (0), less than (1) or ≥30–60 min per week, less than (1) or ≥1–3 h per week (0), less than (1) or ≥3–5 h per week per week (0), less than (1) or ≥5–7 h per week, per week (0), less than (1) or ≥7–9 h per week (0).
Intensity of exposure	Respondents were considered to be at a higher risk of poor mental health if neighbourhood levels of vegetation cover were not met: less than (1) or ≥10% (0), less than (1) or ≥15% (0), less than (1) or ≥20% (0), less than (1) or ≥25%, less than (1) or ≥30% (0), less than (1) or ≥35% (0).

4. Data Availability

Due to third party restrictions, the data are available on request from the corresponding author. The dataset will be available from the NERC Environmental Data Information Centre from mid-2017.

References

1. Lovibond, S.H.; Lovibond, P.F. *Manual for the Depression Anxiety Stress Scales*; Psychology Foundation: Sydney, Australia, 1995.
2. Altshuler, B. Modeling of dose-response relationships. *Environ. Health Perspect.* **1981**, *42*, 23–27.
3. Rueckinger, S.; von Kries, R.; Toschke, A.M. An illustration of and programs estimating attributable fractions in large scale surveys considering multiple risk factors. *BMC Med. Res. Methodol.* **2009**, doi:10.1186/1471-2288-9-7.
4. Sampson, R.J.; Raudenbush, S.W.; Earls, F. Neighborhoods and violent crime: A multilevel study of collective efficacy. *Science* **1997**, *277*, 918–924.
5. Sampson, R.J.; Morenoff, J.D.; Earls, F. Reciprocated Exchange. Avaliable online: http://dcyfernetsearch.cehd.umn.edu/sites/default/files/PsychometricsFiles/Sampson-Reciprocated%20Exchange%20(Ages%2018-older).pdf (accessed on 5 March 2015).
6. Bullen, P.; Onyx, J. *Measuring Social Capital in Five Communities in NSW—A Practitioner's Guide*; Centre for Australian Community Organisations and Management: Coogee, Australia, 1998.

7. Liang, S. *Quantitative Remote Sensing of Land Surfaces*; Kong, J.A., Ed.; John Wiley & Sons, Inc.: Hoboken, NY, USA, 2004.
8. LAStools, "Efficient LiDAR Processing Software" (Version 141017, Academic). Available online: http://rapidlasso.com/LAStools (accessed on 8 May 2016).
9. Hancock, S.; Disney, M.; Muller, J.-P.; Lewis, P.; Foster, M. A threshold insensitive method for locating the forest canopy top with waveform lidar. *Remote Sens. Environ.* **2011**, *115*, 3286–3297.
10. Quantum GIS Development Team. Quantum GIS Geographic Information System v2.6. Open Source Geospatial Foundation Project. 2016. Available online: http://qgis.osgeo.org (accessed on 8 May 2016).
11. R Development Core Team. R: A Language and Environment for Statistical Computing. Vienna, Austria: R Foundation for Statistical Computing. 2016. Avaliable online: http://R−project.org (accessed on 8 May 2016).
12. Australian Bureau of Statistics. Mental Health. Available online: http://www.abs.gov.au/AUSSTATS/abs@.nsf/Lookup/4102.0Main+Features30March%202009 (accessed on 16 April 2015).
13. Osborn, D.P.J. The poor physical health of people with mental illness. *West. J. Med.* **2001**, *175*, 329–332.

© 2017 by the authors; licensee MDPI, Basel, Switzerland. This article is an open access article distributed under the terms and conditions of the Creative Commons by Attribution (CC-BY) license (http://creativecommons.org/licenses/by/4.0/).
